# Global Genome Response of *Escherichia coli* O157∶H7 Sakai during Dynamic Changes in Growth Kinetics Induced by an Abrupt Downshift in Water Activity

**DOI:** 10.1371/journal.pone.0090422

**Published:** 2014-03-03

**Authors:** Chawalit Kocharunchitt, Thea King, Kari Gobius, John P. Bowman, Tom Ross

**Affiliations:** 1 Food Safety Centre, Tasmanian Institute of Agriculture, University of Tasmania, Hobart, Tasmania, Australia; 2 Commonwealth Scientific and Industrial Research Organisation Animal, Food and Health Sciences, North Ryde, New South Wales, Australia; 3 Commonwealth Scientific and Industrial Research Organisation Animal, Food and Health Sciences, Werribee, Victoria, Australia; Indian Institute of Science, India

## Abstract

The present study was undertaken to investigate growth kinetics and time-dependent change in global expression of *Escherichia coli* O157∶H7 Sakai upon an abrupt downshift in water activity (a_w_). Based on viable count data, shifting *E. coli* from a_w_ 0.993 to a_w_ 0.985 or less caused an apparent loss, then recovery, of culturability. Exponential growth then resumed at a rate characteristic for the a_w_ imposed. To understand the responses of this pathogen to abrupt osmotic stress, we employed an integrated genomic and proteomic approach to characterize its cellular response during exposure to a rapid downshift but still within the growth range from a_w_ 0.993 to a_w_ 0.967. Of particular interest, genes and proteins with cell envelope-related functions were induced during the initial loss and subsequent recovery of culturability. This implies that cells undergo remodeling of their envelope composition, enabling them to adapt to osmotic stress. Growth at low a_w_, however, involved up-regulating additional genes and proteins, which are involved in the biosynthesis of specific amino acids, and carbohydrate catabolism and energy generation. This suggests their important role in facilitating growth under such stress. Finally, we highlighted the ability of *E. coli* to activate multiple stress responses by transiently inducing the RpoE and RpoH regulons to control protein misfolding, while simultaneously activating the master stress regulator RpoS to mediate long-term adaptation to hyperosmolality. This investigation extends our understanding of the potential mechanisms used by pathogenic *E. coli* to adapt, survive and grow under osmotic stress, which could potentially be exploited to aid the selection and/or development of novel strategies to inactivate this pathogen.

## Introduction

Enterohemorrhagic *Escherichia coli* (EHEC) O157∶H7 have emerged as important food-borne pathogens of considerable public health concern. They can cause a range of human illnesses including diarrhea, hemorrhagic colitis, and the life-threatening hemolytic uremic syndrome [1], [2]. The majority of reported outbreaks and sporadic cases of O157∶H7 infection appear to be attributed to the consumption of foods of bovine origin, although cases involving dairy products, water, vegetables and fruit products have also been reported. Numerous studies have also identified ruminant animals, especially cattle, as the major reservoir of *E. coli* O157∶H7, which is usually found in the faeces and rumen, on the hide and derived carcass surfaces [1]–[3].

Increasing the osmotic pressure (lowering a_w_) is one of the most widely used methods in food preservation to control the growth of bacteria, including *E. coli*. Reduction in the external a_w_ typically results in a rapid loss of the cytoplasmic volume in a process called plasmolysis, and causes reduced respiration and growth arrest, whereas both intracellular ATP and cytoplasmic pH have been reported to increase [4], [5]. To adapt to hyperosmotic stress, bacteria employ adaptive mechanisms referred to generally as osmoregulatory systems. A major role of these systems is to maintain the proper intracellular osmotic pressure within tolerable limits. This generally involves accumulation of charged solutes (e.g. potassium ions (K^+^) and glutamate), followed by accumulation of compatible solutes either through *de novo* biosynthesis (e.g. trehalose) or through uptake from the external environment (e.g. glycine betaine and proline) [6], [7]. Furthermore, it is well established that bacterial cells previously exposed to osmotic stress (i.e. osmotically-adapted cells) acquire increased resistance to other stresses such as high temperature and oxidative stresses [6], [8]. Therefore, the ability of pathogenic bacteria to adapt to and survive under adverse conditions could increase the risk of food-borne illness. A detailed understanding of how *E. coli* O157∶H7 adapts to hyperosmotic stress could aid in identification of potential targets and develop effective interventions for controlling or eliminating this pathogen.

Previously, we employed both cDNA microarray and 2D-LC/MS/MS analyses to elucidate the genome and proteome expressions of exponential phase *E. coli* O157∶H7 strain Sakai grown under steady-state conditions, relevant to low temperature and water activity conditions experienced during carcass chilling [9]. It was found that *E. coli* O157∶H7 respond to these steady-state conditions, including osmotic stress (25°C a_w_ 0.967) by activating the master stress response regulator RpoS and the Rcs phosphorelay system involved in the biosynthesis of the exopolysacharide colanic acid, as well as down-regulating genes and proteins involved in chemotaxis and motility [9]. Such findings have provided a baseline of knowledge of the potential molecular mechanisms enabling growth of this pathogen under these stress conditions. To gain a deeper insight into the physiology of exponentially growing *E. coli* O157∶H7 Sakai in response to hyperosmolality, the present study investigated the growth kinetics of this pathogen subjected to sudden osmotic upshift (from a_w_ 0.993 to a_w_ 0.980, 0.975, 0.970, 0.967 or 0.960), as well as to examine the time-dependent alterations in its transcriptome and proteome upon hyperosmotic shock from a_w_ 0.993 to a_w_ 0.967 at a constant temperature of 35°C. The global responses were analyzed by both cDNA microarray and 2D-LC/MS/MS analyses. Differences in gene and protein expression patterns in *E. coli* before and after osmotic shock were analyzed through quantitative and comparative analysis of time-series changes in both mRNA and protein levels.

## Materials and Methods

### Division of experimental work

Parallel transcriptomic and proteomic studies were undertaken in two independent laboratories, CSIRO Animal, Food and Health Sciences (North Ryde, Australia) and Food Safety Centre (Hobart, Australia), respectively. A series of experiments on the growth response of *E. coli* O157∶H7 to hyperosmotic shifts were also conducted in the Food Safety Centre (Hobart, Australia). Although conducted in separate laboratories, the protocol was identical for the hyperosmotic shock experiments with the only variation involving the different methods used for extraction of mRNA and proteins, as well as the use of different equipment for optical density measurement (Transcriptomic: Pharmacia Biotech Novaspec II spectrophotometer; and Proteomic: Spectronic 20, Bausch and Lomb) and waterbaths for incubation of cultures (Transcriptomic: Julabo SW23 waterbath (80 rpm); and Proteomic: Ratek SWB20D shaking waterbath (80 oscillations/min)).

### Bacterial strain and preparation of inocula

*E. coli* O157∶H7 strain Sakai [10], [11] obtained from Dr. Carlton Gyles (Guelph, Canada) was used in this study. The strain was maintained at −80°C. Prior to any experiments, the culture was revived by plating onto brain-heart infusion (BHI) agar (Oxoid, CM225), and incubating at 37°C for 24 h. A well-isolated single colony was aseptically transferred into 25 ml of BHI broth. After incubation at 37°C for 20 h, the broth culture was stored at 4°C and used as a ‘working’ culture within a week.

### Preparation of low a_w_ broth

A BHI broth containing a very high concentration of NaCl was prepared to be used in osmotic upshift experiments. Specifically, an appropriate amount of NaCl was added to fresh BHI broth (a_w_ 0.993±0.003) to achieve the final a_w_ of 0.760 (±0.003), as determined from the Tables of Chirife and Resnik [12]. This NaCl-concentrated BHI broth was then sterilized (121°C, 20 min) and kept at room temperature until used. The final a_w_ of each NaCl-modified BHI broth was confirmed using an Aqualab CX-2 dew point instrument (Decagon Devices, Inc., Pullman, USA).

### An abrupt downshift in water activity

An appropriate volume of the working culture was diluted 1∶10^4^ in 25 ml of pre-warmed (35°C) BHI broth. This ‘primary’ culture was incubated at 35°C in a water bath with agitation (80 oscillations/min; Ratek Instruments, Boronia, Australia), and its growth monitored turbidimetrically at 600 nm with a Spectronic 20 spectrophotometer (Bausch and Lomb, Rochester, USA). After achieving an OD_600_ of 0.1±0.01 (i.e. the mid-exponential phase of growth, ∼10^7^ CFU/ml), a 1∶10^2^ dilution of the primary culture was prepared further in a 25 ml of pre-warmed (35°C) BHI broth to prepare a ‘secondary’ culture. This culture was then re-incubated at 35°C with agitation until it reached the mid-exponential growth phase (OD_600_ of 0.1±0.01). At this point, after removing an aliquot for subsequent analysis, an appropriate volume of the NaCl-concentrated BHI previously prepared (pre-warmed to 35°C) was added to the culture to create a series of hyperosmotic broths at a_w_ 0.980, 0.975, 0.970, 0.967 or 0.960. Each of these inoculated a_w_-modified broths was re-incubated at 35°C with shaking.

### Microbiological analysis

*E. coli* growth in each a_w_-modified broth was monitored by viable count method. At regular intervals, 0.1 ml of the test broth was obtained and serially diluted in 0.1% peptone water (PW; Oxoid, LP0037) containing 0.85% NaCl. Aliquots of the appropriate dilution were then plated onto BHI agar supplemented with 0.1% sodium pyruvate (Sigma, P8574) using an Autoplate 4000 spiral plater (Spiral Biotech, Bethesda, USA). All plates were then incubated at 37°C for 24 h before colonies were counted. All growth experiments were replicated at least twice, and the results reported as the average of the replicates.

Log_10_ CFU/ml was plotted against time to generate growth curve for each osmotic treatment. All growth curves were analyzed using linear regression to estimate generation time (GT) and lag time (LT), according to Mellefont et al. [13]. Briefly, a straight line was fitted to the data points (i.e. log_10_ CFU/ml) that best represent exponential phase of growth. To ensure the accuracy of this estimation, at least four time points over this growth phase were used, and the minimum acceptable R^2^ value for the regression line was 0.95. GT was then calculated by dividing 0.301 (equivalent to log_10_ 2) by the slope of the line. LT was estimated from the time at which the regression line through the exponential part of the growth curve first exceeded the initial cell numbers after applying the hyperosmotic shift (i.e. the time taken to increase above starting numbers). We also determined the time taken for cells to resume exponential growth following the shift. This is referred to as ‘adaptation time’ (AT), and was estimated visually from the growth curves. To measure the amount of *work* to be done by *E. coli* to adjust to new conditions, relative lag time (RLT) was calculated by dividing LT or AT by GT [14], [15].

### Preparation of samples for transcriptomic and proteomic analyses

In a separate set of hyperosmotic shift experiments, a rapid downshift of *E. coli* cultures from a_w_ 0.993 to a_w_ 0.967 was carried out as described above to prepare samples for cDNA microarray and 2D-LC/MS/MS analysis. An appropriate volume of cell cultures was harvested before osmotic upshift (i.e., control culture or non osmotically-treated cells), and at 0 (i.e., immediately after the shift), 30, 80, and 310 min after the shift. Independent samples were then subjected to extraction of RNA, and both soluble and membrane proteins, according to the methods of Kocharunchitt et al. [9]. It also should be noted that the current proteomic study used the same control samples (i.e., at 35°C a_w_ 0.993) as in the companion study [16]. The number of biological replicates performed for each time point in the transcriptomic and proteomic analyses is described in Table 1.

**Table 1:** Summary of the number of protein identifications and the number of differentially expressed genes and proteins during exposure of *E. coli* O157∶H7 Sakai to hyperosmotic stress.

	Number of biological replicates		Number of proteins			Number of differentially expressed elements^d^		
Time points	Transcriptome	Proteome^a^	Membrane fraction^b^	Soluble fraction^b^	Total fraction^c^	Transcriptome (total)^e^	Proteome (total)	Transcriptome *vs.* Proteome
Before a_w_ downshift	3	6(6)	343	1,281	1,225	NA^f^	NA	NA
Immediately after shift	3	2(2)	710	865	1,020	3(5)	158(30)	0(0)
30 min after shift	3	2(2)	649	901	1,064	182(15)	162(21)	2(0)
80 min after shift	3	2(2)	615	904	968	772(414)	126(37)	16(8)
310 min after shift	3	2(2)	1,071	1,259	1,416	605(326)	399(12)	145(9)

a Number of replicates performed for soluble (outside brackets) and membrane (within brackets) fractions of *E. coli*.

b Number of protein identifications passing the filtering criteria (i.e. at PeptideProphet and ProteinProphet of ≥0.9) in membrane and soluble fractions of the *E. coli* proteome.

c Number of protein identifications with high confidence in total fraction of the *E. coli* proteome.

d Number of elements with increased (outside brackets) and decreased (within brackets) expression.

e Analysis does not include the number of differentially expressed undefined intergenic regions in the transcriptome.

f NA; not applicable.

Both transcriptomic (cDNA microarray) and proteomic (2D-LC/MS/MS) analyses were carried out as described by Kocharunchitt et al. [9].

### Microarray data analysis

Gene expression was analyzed using GeneSpring GX 10.0 software (Agilent Technologies, Palo Alto, CA). Array data was normalized using the Robust Multi-Array Analysis (RMA) algorithm [17]. Probesets with an intensity value in the lowest 20th percentile among all the intensity values were excluded. The remaining probesets, with intensity values between the 20th and 100th percentile, were used for downstream statistical analysis. Significance analysis was conducted using one way analysis of variance (ANOVA) using the multiple correction testing method of Benjamini and Hochberg [18] with a *P*-value cut-off of <0.01. Genes were considered to be differentially expressed if the fold change was >2.

### MS/MS data analysis

MS/MS data obtained from each protein sample of membrane and soluble fractions were processed by the Computational Proteomics Analysis System (CPAS), a web-based system built on the LabKey Server (v9.1, released 02.04.2009) [19]. The experimental mass spectra produced were subjected to a semi-tryptic search against the combined databases of *E. coli* O157∶H7 Sakai (5,318 entries in total) downloaded from the National Center for Biotechnology Information (NCBI, https://www.ncbi.nlm.nih.gov/, downloaded 25.11.2008) using X!Tandem v2007.07.01 [20]. These databases included the *E. coli* O157∶H7 Sakai database (5230 entries, NC_002695.fasta) and two *E. coli* O157∶H7 Sakai plasmid databases, plasmid pO157 (85 entries, NC_002128.fasta) and plasmid pOSAK1 (three entries, NC_002127.fasta). The parameters for the database search were as follows: mass tolerance for precursor and fragment ions: 10 ppm and 0.5 Da, respectively; fixed modification: cysteine cabamidomethylation (+57 Da); and no variable modifications. The search results were then analyzed using the PeptideProphet and ProteinProphet algorithms from the Trans Proteomic Pipeline v3.4.2 [21], [22]. All peptide and protein identifications were accepted at PeptideProphet and ProteinProphet of ≥0.9, corresponding to a theoretical error rate of ≤2% [21]. To assess a false-positive discovery rate for each dataset, the MS/MS spectra were searched against the target-reversed (decoy) database using the same search criteria as described above. The false-positive discovery rate of peptide identifications was then estimated by dividing the number of spectra matching decoy peptides with the total number of spectra, and appeared to be less than 5% for all datasets (Table S1 in File S1).

All protein identifications that passed the above criteria were further assessed based on the confidence level of protein identifications across biological replicates of each fraction of *E. coli* at each time point. The confidence level was based on the number of unique peptides identified from one sample and the number of replicates in which the protein was detected. Specifically, proteins identified by more than one unique peptide in at least one of the replicates were considered to have a ‘high’ confidence score. The ‘intermediate’ confidence level was assigned to proteins with a single peptide hit that was detected in more than one replicate. ‘Low-confidence’ proteins were considered to be those identified by a single unique peptide and found in only one replicate. Only protein identifications with a ‘high’ and ‘intermediate’ confidence level (referred to as having a ‘high confidence score’) were accepted for further analysis.

Following data filtering, all protein identifications with high confidence from membrane and soluble fractions of the same sample were combined to represent a ‘total’ proteome of the corresponding sample. This combined approach was carried out to improve reliability of the proteomic data. The R^2^ values, as determined by linear regression analysis indicated a relatively stronger linear correlation between spectral counts (SpCs, the number of MS/MS spectra) for all possible pair-wise comparisons of replicates of pooled fractions (average R^2^ = 0.89±0.06) when compared to that of replicates of each fraction (average R^2^ = 0.64±0.20, and 0.85±0.06 for membrane and soluble fractions, respectively). Each of the total fractions across the biological replicates was then used to generate the final list of proteins considered to be representative of each time point of sampling.

### Protein abundance ratio and its significance

Spectral count generated by 2D-LC/MS/MS analysis was used as a semi-quantitative measure of protein abundance [21]. The total abundance of each protein in a sample was obtained by pooling spectral counts detected for a given protein from membrane and soluble fractions of the same sample (i.e. the same biological replicate). Normalized spectral abundance factor (NSAF) was then applied to the pooled spectral count of each protein, according to Zybailov et al. [23]. Briefly, the NSAF for a given protein is the pooled spectral count of the protein divided by its amino acid length (L), and normalized against the average SpC/L across all proteins in the corresponding dataset. The NSAF values were then averaged across all biological replicates at each time point of sampling to obtain the representative values of NSAF.

Fold changes in protein abundance due to hyperosmotic shock were calculated as the log_2_ ratio of the average NSAF value in the osmotically-treated sample over the average NSAF value of the control. To avoid errors during fold change calculation in cases where a zero value was obtained, a correction factor of 0.01was added to each of the average NSAF values. Furthermore, differences in protein abundance were statistically analyzed using the beta-binomial test implemented in R [24]. All proteins with a *P*-value of <0.01 and at least 2 fold change were considered to be differentially abundant.

### Transcriptomic and proteomic data mining

Information on identified genes and proteins such as protein and gene names, ECs numbers (locus tag), GI numbers, NCBI Reference Sequence (RefSeq), protein sizes and molecular masses were obtained from the UniProt knowledgebase (http://www.uniprot.org/, accessed on 5.12.2010) and the NCBI database (accessed on 5.12.2010). Preliminary functions and properties of the genes and proteins were based on the EcoCyc database (http://www.ecocyc.org/, accessed on 13.12.2010), and the Kyoto Encyclopedia of Genes and Genomes (KEGG) database (http://www.genome.jp/kegg/, accessed on accessed on 4.12.2010). Protein or gene names in conjunction with ECs numbers were used as a unique identifier for genes and/or proteins.

### Predefined set enrichment analysis

To determine changes in overall expression of a predefined set of genes or proteins due to osmotic upshift, a *t*-test based method of Boorsma et al. [25], known as T-profiler, was performed on the log_2_ expression ratios. The predefined sets of genes or proteins used for T-profiler analysis were based on the functional role categories and/or metabolic pathways of the following databases: the JCVI Comprehensive Microbial Resource (JCVI CMR) database (http://cmr.jcvi.org/cgi-bin/CMR/CmrHomePage.cgi, accessed on 5.12.2010), the EcoCyc database collection (http://www.ecocyc.org/, accessed on 13.12.2010), and the KEGG database (http://www.genome.jp/kegg/, accessed on 4.12.2010), as well as based on the lists of genes or proteins previously known to be positively controlled by major regulons, including CpxRA [26], RpoE [27], RpoH [28], RpoS [29]–[31], and the Rcs phosphorelay system [32]. The *T*-value obtained from the analysis was determined only for sets that contained at least five members, and its significance was established by using the associated two-tailed *P*-value. All predefined sets with a *P*-value less than 0.1 were considered to be statistically significant.

### Clustering analysis of transcriptomic and proteomic data

To determine similarities in transcriptomic and proteomic profiles of *E. coli* during exposure to a sudden downshift in a_w_, hierarchical clustering analysis (HCA) was performed on the *T*-values calculated for JCVI CMR functional categories. The T-profiler results for the transcriptome and proteome of *E. coli* during steady-state growth at 25°C a_w_ 0.967 (data of Kocharunchitt et al. [9]) were also included in the clustering analysis for comparison. The degree of similarity was calculated with the Euclidean distance metric and complete linkage as a clustering method, using Cluster 3.0 software [33]. The clustering result was then visualized in TreeView version 1.1.3 [34].

### Transcriptomic and proteomic data accession numbers

The transcriptomic data are available in the ArrayExpress database (www.ebi.ac.uk/arrayexpress) [35] under accession number E-MTAB-2011. The mass spectrometry proteomic data are deposited to the ProteomeXchange Consortium (http://proteomecentral.proteomexchange.org) via the PRIDE partner repository [36] with the dataset identifiers PXD000583 and PXD000541.

## Results and Discussion

### Growth of *E. coli* O157∶H7 Sakai upon a_w_ downshift

**Figure 1:**
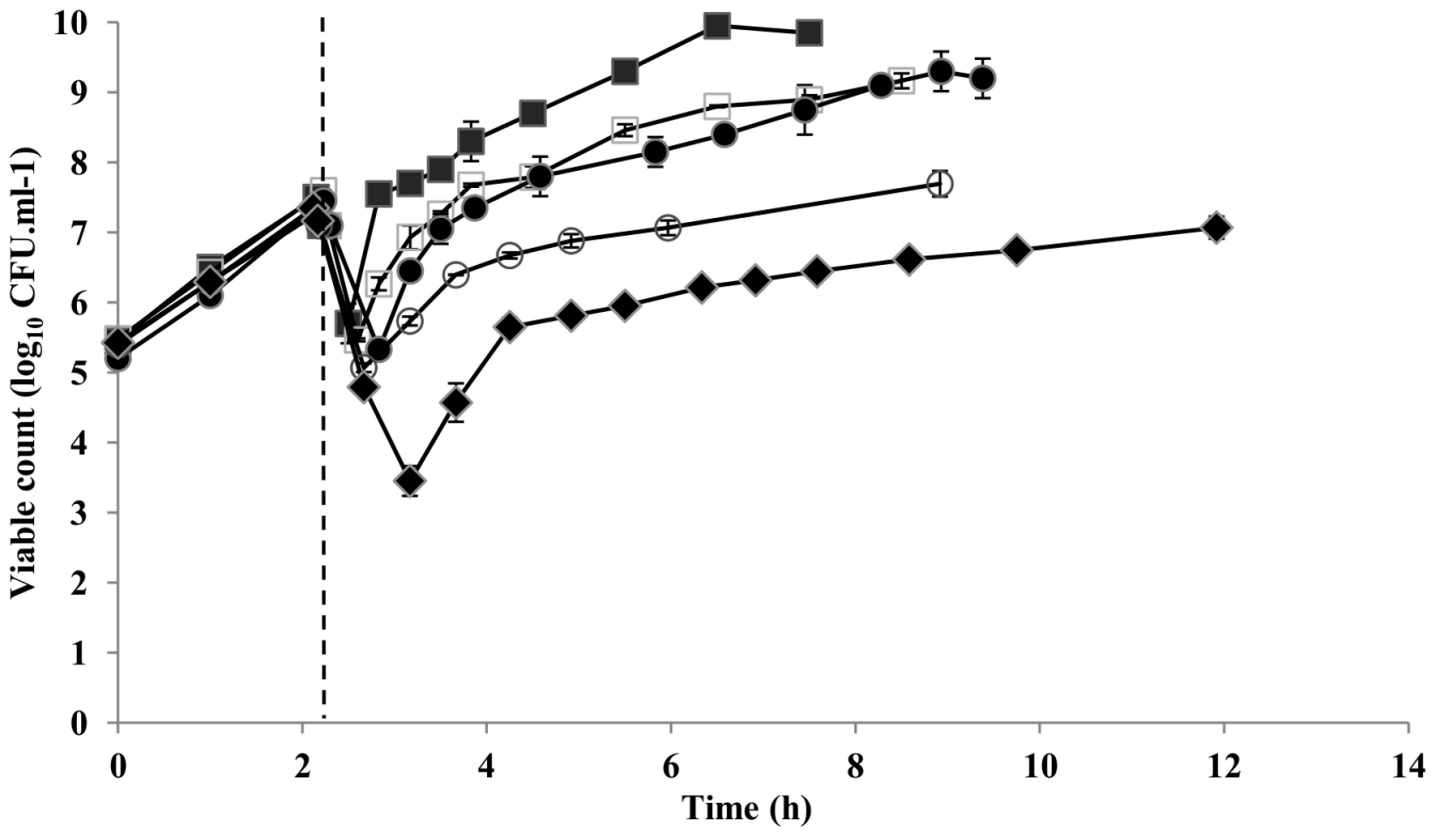
Growth of *E. coli* O157∶H7 upon water activity downshift. Growth response of *E. coli* was determined by viable count when subjected to a rapid downshift from a_w_ 0.993 to a_w_ 0.980 (▪), 0.975 (□), 0.970 (•), 0.967 (○) and 0.960 (♦) at a constant temperature of 35°C. The time at which hyperosmotic shift was applied is indicated by a dotted line. Data points represent means ± standard deviations of at least two independent replicates.

Growth of exponential phase *E. coli* (OD_600_ of ∼0.1±0.01) was assessed in a nutrient rich medium when subjected to a rapid downshift from a_w_ 0.993 to a_w_ 0.980, 0.975, 0.970, 0.967 and 0.960 at a constant temperature of 35°C. All downshifts resulted in a growth interruption before cells resumed their growth at a slower rate (Figure 1). Estimates of kinetic parameters for each a_w_ downshift are summarized in Table 2. The generation times obtained were consistent with those expected from the predictive model of Ross et al. [37], and both generation and lag times increased with respect to the magnitude of the osmotic shift. The RLT_LT/GT_ response of *E. coli* increased in a linear manner (y = −225.64x−1.84, R^2^ = 0.89) with the magnitude of the shift. This is consistent with previous studies, reporting that cells shifted to progressively harsher conditions require more adaptation work to be done, i.e. RLT become larger [13], [38], [39].

**Table 2:** Summary of the growth kinetics of exponential phase *E. coli* O157∶H7 Sakai subjected to a sudden downshift in water activity from a_w_ 0.993 to a_w_ 0.980, 0.975, 0.970, 0.967 and 0.960, as determined by viable count.

				Relative lag time (RLT)	
Water activity downshift	Generation time, GT (h)	Lag time, LT (h)^a^	Adaptation time, AT (h)^b^	LT/GT	AT/GT
a_w_ 0.980	0.45	0.69	0.68	1.53	1.51
a_w_ 0.975	0.67	1.55	0.94	2.31	1.40
a_w_ 0.970	0.73	2.04	1.27	2.79	1.74
a_w_ 0.967	1.41	4.74	1.53	3.36	1.09
a_w_ 0.960	1.68	10.57	2.13	6.29	1.27

a Lag time is calculated by linear regression as the time taken to increase above starting numbers.

b Adaptation time is estimated visually as the time taken for cells to resume exponential growth.

*E. coli* appeared to have a complex pattern of growth in response to sudden downshifts in a_w_. A rapid, but transient decrease in bacterial numbers was observed immediately after a shift to lower a_w_ (Figure 1). This indicates that cells might lose their culturability on enumeration media, possibly due to injury. The degree of loss of culturability also became progressively larger with the larger shifts in a_w_, although this loss of culturability occurred at a similar rate for all a_w_ tested. However, the loss of culturability was reversed within the lag period at a rate faster than that characteristic of exponential growth under the given conditions (Figure 1). Furthermore, it was evident that during treatment at a_w_ 0.967 and 0.960, the exponential growth occurred before the ‘lag period’ (when defined as the time taken to increase above starting numbers) was resolved. This suggests that cell death occurred upon the shift at the two lowest a_w_ tested. A similar injury and recovery phenomenon has been reported in other studies following the application of sub-lethal stressful treatments [40]–[42]. However, our results are in contrast to the study of Mellefont et al. [38] in which abrupt osmotic shifts of *Salmonella* Typhimurium caused inactivation (rather than loss of culturability) of a sub-population followed by renewed exponential growth.

The complexity of the response of *E. coli* to osmotic upshifts (i.e. irregularly shaped growth curves arising from the initial decline and subsequent recovery in cell numbers) may contribute to unusual estimates of RLT, although a consistent and reproducible pattern of the RLT_LT/GT_ response was obtained in this study. Death of cells would mask the time when the lag period is actually resolved, resulting in overestimation of lag time. This led us to determine adaptation time (AT), i.e. the time taken for bacterial cells to resume exponential growth after hyperosmotic shift. The adaptation times increased with the magnitude of the osmotic shift, a similar trend to that observed in lag times (Table 2). However, when RLT estimates were calculated using the adaptation time, there was no systematic variation in RLTs_AT/GT_ over the a_w_ range tested (y = 14.53x+1.73, R^2^ = 0.20). This implies that surviving *E. coli* cells require a similar amount of *work* to be done to adapt to a given decrease in a_w_ and, thus, is inconsistent with the observed pattern of RLT_LT/GT_ response and previous studies in which shifts to progressively harsher environments would require more adaptation *work* to be done [13], [38], [39]. Further investigations at more extreme osmotic upshifts together with validation of our data by culture-independent methods are necessary to confirm the present observations.

### Molecular response of *E. coli* O157∶H7 Sakai to hyperosmotic shock

To understand better the potential mechanisms adopted by *E. coli* to respond to osmotic shock and hyperosmotic stress, the present study employed transcriptomic and proteomic analyses in parallel in independent experiments to examine temporal alterations in mRNA expression and protein production in *E. coli* upon a rapid downshift from a_w_ 0.993 to a_w_ 0.967 at a constant temperature of 35°C. Bacterial cells were harvested before hyperosmotic shift, and 0 (i.e. immediately after the shift), 30, 80, and 310 min after the shift (Figure 1). It also should be noted that these time points were chosen with the aim to characterize the physiology of *E. coli* during dynamic changes in growth kinetics induced by hyperosmotic shift. Specifically, the samples taken at time 0 and 30 min were obtained during the period in which the loss of cell culturability was observed, whereas the samples at time 80 and 310 min respectively reflected the physiological state of *E. coli* during the ‘recovery’ period and during growth after the shift. Independent samples were processed for mRNA, and membrane and soluble protein extractions as appropriate for cDNA microarray and 2D-LC-MS/MS analyses, respectively. By using multidimensional LC/MS/MS analysis together with data filtering, protein identifications with high confidence were obtained for total fractions of *E. coli* at each time point (Table 1).

Changes in gene expression and protein abundance due to hyperosmotic stress were determined by comparing the profiles obtained after the shift to that of a reference sample taken prior to the shift. Table 1 describes the number of genes and proteins with significant changes in expression level at each time point. It was found that the number of up-regulated elements (referred to here as genes and proteins) was dominant over those with decreased expression level. A complete list of these genes and proteins with their comprehensive annotations, as well as their comparison to previously published studies is shown in Table S2 in File S1.

Exposure of *E. coli* to a rapid downshift in a_w_ evokes a highly complex regulatory process involving expression changes of different groups of genes and proteins, which were condensed using the T-profiler analysis (see Tables S3–S5 in File S1). These genes and proteins are involved in a number of functional categories and metabolic pathways, as well as play an important role in the stress responses that are controlled by regulons, as discussed below.

### Comparison of transcriptome and proteome

To determine the level of correlation between the transcriptomic and proteomic data, the list of differentially expressed genes and proteins at each time point was compared. It was found that the level of correlation was low with an average of only 4.32%±7.09% of genes and proteins matched across all time points (Table 1). This was despite that a number of trends were still observed in the data (discussed below). Consistent with these results, several studies have reported poor correlation between gene and protein expression profiles [9], [43], [44]. Several possible explanations for this have also been suggested, including different controlling mechanisms at the transcriptome and proteome levels, post-transcriptional mechanisms affecting the protein translation rate, differences occurring between the half-lives of specific proteins or mRNAs, and the intracellular location and molecular association of the protein products of expressed genes [45].

**Figure 2:**
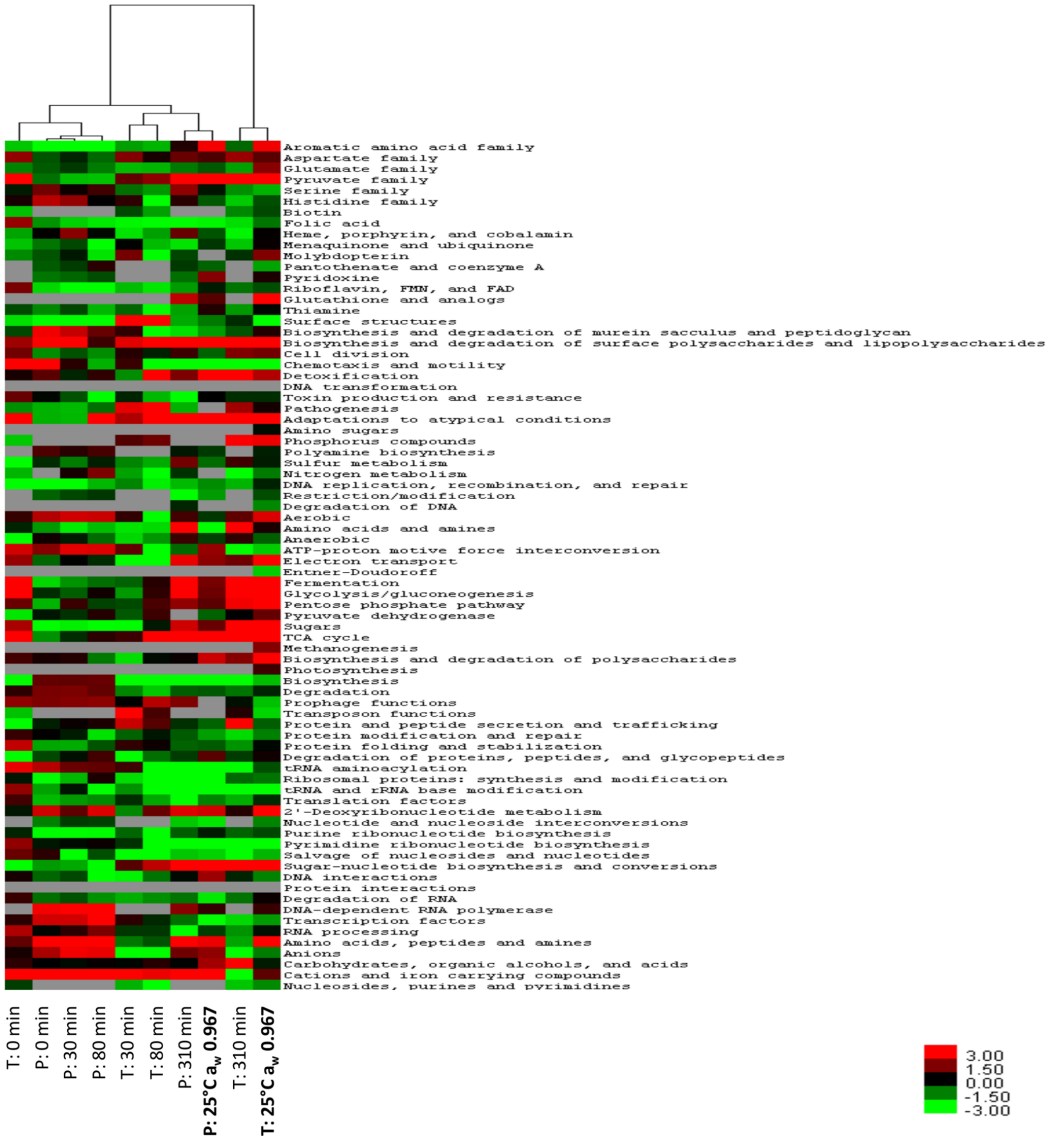
Hierarchical clustering analysis of transcriptomic and proteomic data. The heat map represents the *T-*values calculated for transcriptomic (T) and proteomic (P) profiles based on JCVI CMR functional categories (row), and is linked by a dendrogram representing clustering of these profiles obtained from *E. coli* upon hyperosmotic shift, and those during exponential growth at 25°C a_w_ 0.967 [9] (top). The color code is as follows: red indicates positive *T*-values; green indicates negative *T*-values; and grey indicates not determined.

To further determine the similarity between gene and protein profiles across different time points, the clustering analysis was performed on the T-profiler results obtained in the present study (i.e. both transcriptomic and proteomic data) and that of Kocharunchitt et al. [9] on the transcriptomic and proteomic responses of *E. coli* during exponential growth at a similar osmotic stress condition (25°C a_w_ 0.967) (Figure 2). It was found that gene and protein profiles could be grouped into three major clusters. The first cluster is comprised of the gene profile at 0 min after downshift in a_w_ and the protein profiles within the first 80 min following the shift, whereas the second cluster contains the gene profiles at time 30 and 80 min, and the protein profile at time 310 min and that of steady-state growth at 25°C a_w_ 0.967. For the last cluster, it consists of the gene profile at 310 min and that of steady-state growth at 25°C a_w_ 0.967. These findings, taken together, indicate that the transcriptomic data at a given time point was a reflection of subsequent, rather than concomitant proteomic data, providing a valid explanation for the low level of correlation between gene and protein profiles observed here (see above). The apparent alignment of a given transcriptomic data with proteomic data at a later time point has also led to the suggestion that *E. coli* cells might take longer to adjust their gene expression (i.e. transcription) in response to hyperosmotic shock and that change in the processes of translation and post-translational modification might be the potential mechanism used by bacteria to quickly adapt or respond to such environmental change. In keeping with this, Rossouw et al. [46] have reported a similar observation for the transcriptome and proteome of *Saccharomyces cerevisiae* during alcoholic fermentation. Furthermore, it should be noted that the last two clusters imply that the transcriptome and proteome of *E. coli* at 310 min after the shift were similar to those of *E. coli* grown at high osmolality (25°C a_w_ 0.967). This supports earlier observations that *E. coli* had already established balanced growth typical of growth under such stress.

Despite the discordant alignment of transcript and protein levels observed here, previous reports have indicated the need for complementary profiling of transcriptome and proteome to fully develop an understanding of the functional architecture of genomes and gene networks [47], [48].

### Transcription and translation

Bacteria generally control the cellular content of transcriptional and translational machineries according to their physiological state. Faster-growing cells synthesize proteins more quickly, and the levels of ribosomes and ribosome-associated proteins correlates well with growth rate [49], [50]. Reduced growth rate as a result of environmental conditions including high osmolarlity has an effect on gene expression and protein production [9], [51]. However, the present findings did not show a strong alteration in expression of genes and proteins associated with RNA polymerase in response to a_w_ downshift (Table S4 in File S1). This was despite the transcriptomic analysis revealing a significant down-regulation of *pcnB* encoding poly(A) polymerase I (1.06 log_2_-fold) only at 80 min of the treatment. On the other hand, it was found that the level of translational machinery was affected after prolonged exposure to osmotic stress. Several ribosomal proteins exhibited a decrease in their abundance at 310 min after the treatment, as indicated by a significant negative *T*-value of 4.39 (Table S4 in File S1). This response agrees well with the observations of Kocharunchitt et al. [9] who reported a reduced translational capacity in osmotically-adapted cells.

### Carbohydrate catabolism and energy generation

A sudden downshift in a_w_ was generally found to have no major effect on expression of genes and proteins involved in carbohydrate metabolism during the adaptation period following hyperosmotic shock (i.e. at time 0, 30 and 80 min after the shift). Only three genes encoding the enzymes in the glycolysis/gluconeogenesis pathway exhibited a significant change in transcription level, including *fbaB* (4.60 log_2_-fold increase), *tpiA* (1.03 log_2_-fold decrease) and *ybhA* (1.15 log_2_-fold decrease). On the other hand, when the osmotically-treated cells had resumed exponential growth (i.e. at time 310 min), several genes and proteins of the glycolysis and gluconeogenesis pathways appeared to be up-regulated, although significant changes in their overall expression were not evident in the T-profiler analysis (Table S4 in File S1). These included *fbaB*/FbaAB, *pfkB*/PfkAB, *mdh*/Mdh and *maeB*/MaeB for which the expression level increased by up to 6.40 log_2_-fold. Similarly, up-regulation of a number of genes and proteins involved in the pentose phosphate pathway was evident at this time point. Among these, transaldolase A (*talA*/TalA) and transketolase B (*tktB*/TktB), which are involved in the non-oxidative branch of the pentose phosphate pathway, exhibited a significant up-regulation. Regulation of these genes is also known to be positively controlled by RpoS at the transcriptional level (see below) [31].

*E. coli* typically utilizes three alternative modes for energy generation, namely aerobic respiration, anaerobic respiration and fermentation [52]. It has been shown that the aerobic respiration of *E. coli* upon exposure to hyperosmotic shock is severely inhibited as a consequence of plasmolysis, and that the respiratory activity is recovered during deplasmolysis [53]. In the present study, the transcriptomic and proteomic data were inconsistent with regard to responses related to oxidative phosphorylation (Table S4 in File S1). A number of genes encoding the enzymatic complexes involved in oxidative phosphorylation were down-regulated at 80 min of the treatment, as indicated by a significant negative *T*-value of 2.43. These included genes *nuoKLMN* (1.23 log_2_-fold decrease on average) encoding the membrane components of NADH:ubiquinone oxidoreductase I. By contrast, the proteomic analysis indicated a significant increase in overall abundance of proteins involved in oxidative phosphorylation within the first 80 min. A common set of proteins observed at these time points were the components of NADH:ubiquinone oxidoreductase I (NuoCFI; 2.94 log_2_-fold on average), succinate dehydrogenase (SdhA; 2.77 log_2_-fold on average), and ATP synthase (AtpB; 3.32 log_2_-fold on average). These inconsistent observations from two different methodologies may be in line with those of Gygi et al. [45] who compared mRNA and corresponding protein levels, as discussed earlier. However, *E. coli* appeared to increase aerobic respiration after prolonged exposure to hyperosmotic stress, as indicated by significant positive *T*-values for the TCA cycle and glyoxylate bypass pathways (Table S4 in File S1). The transcriptomic study revealed a significant increase in expression of several genes (*aceAB*, *acnA*, *sdhACD* and *gltA*; 3.49 log_2_-fold on average) encoding the enzymes in the TCA cycle and glyoxylate bypass at 80 and 310 min of the stress, whereas almost all the TCA cycle and glyoxylate bypass-related proteins were found to be significantly up-regulated only at time 310 min in the proteomic analysis (Table S2 in File S1).

Taken together, the increased expression of genes and/or proteins involved in the major processes of carbohydrate catabolism and energy generation at 310 min after the downshift may be to compensate for the reduction in activity of these processes at low a_w_, or may reflect a high level of energy production together with an increase in level of precursors for the biosynthesis of various macromolecules. The latter corresponds well with the observation that *E. coli* resumed their growth at or before this time point (Figure 1). Our previous study also indicated an increase in metabolic activities to meet an energy requirement during exponential growth at high osmolality (25°C a_w_ 0.967) [9].

### Two-component regulatory system

The mechanisms involved in osmosensing and subsequent regulatory responses in *E. coli* are strongly controlled by two-component regulatory systems [6], [54]. The EnvZ/OmpR system regulates the transcription of genes encoding porins OmpC and OmpF in response to changes in medium osmolality. This regulation is characterized by an altered ratio of these porins, whereby the synthesis of OmpC increases, and that OmpF decreases under hyperosmotic stress [55], [56]. However, we did not observe a strong activation of this system (Table S2 in File S1), although a significant increase in the abundance of OmpC and OmpR was observed at time 0 and 310 min of the osmotic stress, respectively.

### Amino acids biosynthesis

Biosynthetic genes and proteins for various amino acids had altered expression levels after an abrupt downshift in a_w_ (Tables S2 and S3 in File S1). Among these, the mRNA level of *his*CDF, which encode key enzymes involved in histidine biosynthesis, was significantly reduced at 80 min of the shift (up to 1.14 log_2_-fold). This was accompanied by the observation based on the T-profiler analysis that a significant decrease in the overall expression of histidine biosynthetic genes occurred at this time (Table S3 in File S1). The biosynthesis of histidine is known to be an energetically expensive process, requiring 41 ATP molecules per histidine molecule made [57]. Previous studies have also demonstrated that constitutive expression of the *his* operon caused a number of physiological changes in *E. coli*, including growth inhibition in high-salt media [58], [59]. Therefore, the apparent decrease in the activity of the histidine biosynthetic pathway might imply that *E. coli* suppresses this biosynthetic pathway to prevent an inappropriate use of the cellular energy, possibly for re-allocating the energy to repair cell damage as observed during the recovery period (i.e. at time 80 min) (Figure 1).

On the other hand, the present study revealed an induced activity of the methionine biosynthetic pathway in osmotically-adapted cells (i.e. at 310 min of the treatment), as indicated by a significant up-regulation of *metEH* genes and MetBEH proteins (an increase in abundance of methionine transporters was also observed, see below). Methionine is required for initiation and elongation of proteins, biosynthesis of polyamines, purines and pyrimidines, and various methylation reactions [60], [61]. A previous study has also demonstrated that the growth of *E. coli* at elevated temperatures is limited by the availability of endogenous methionine [60]. This has led to the suggestion that an increase in the cellular level of methionine might contribute to the maintenance of balanced growth rate of *E. coli* at high osmolality. However, Kocharunchitt et al. [9] did not find up-regulation of the methionine biosynthetic pathway during growth at a similar osmotic stress condition of 25°C a_w_ 0.967. This might be due to the difference in the conditions tested in this study (i.e. 35°C a_w_ 0.967). Furthermore, the T-profiler analysis revealed a significant increase in overall expression of genes and proteins responsible for the biosynthesis of amino acids in the pyruvate family at the time point at which osmotically-treated cells resumed their growth after osmotic upshift (i.e. at time 310 min) (Table S3 in File S1). Among these, branched-chain amino acids biosynthetic pathway genes and proteins (*ilvBDG*/IlvBDG) exhibited a significant up-regulation. This agrees well with the observation on the adaptive response of *E. coli* to osmotic stress (25°C a_w_ 0.967) [9]. Taken together, the apparent increase in expression of several genes and proteins related to the amino acid biosynthetic pathways (i.e. methionine, isoleucine and valine) supports the previous hypothesis that an increase in intracellular contents of these amino acids may be part of the responses to enhance growth of *E. coli* under osmotic stress [9]. Horinouchi et al. [62] have also shown that supplementing culture media with specific amino acids (i.e. tryptophan, histidine and branched-chain amino acids) promoted the growth of *E. coli* under another type of stress (i.e. ethanol stress).

### Transport functions

Accumulation of compatible solutes through uptake from the external environment by active transport is one of the most important responses for bacteria to counter hyperosmotic stress [7]. Indeed, the present study found a significant increase in activity of a number of transporters for compatible solutes upon exposure to osmotic upshift (Table S2 in File S1). Although the high-affinity potassium ion (K^+^) transport system (Kdp) did not change in its level after the shift, abundance of the low-affinity system Kup, was up-regulated by up to 3.57 log_2_-fold at all time points. The potassium channel protein Kch also exhibited an increase in its abundance at time 80 min and 310 min (2.55 log_2_-fold on average). Correspondingly, the expression of genes (*gltJI*) encoding transporters for glutamate (the potassium ion counter ion) was found to be induced at these time points (1.47 log_2_-fold on average). Other transport systems that were up-regulated in both transcriptomic and proteomic analyses included those responsible for the uptake of glycine betaine/proline (*proVWX* operon; at 30, 80 and 310 min of the treatment) and other osmoprotectants (*yehWXYZ* operon; at 80 and 310 min). In addition, NhaAB sodium ion/proton antiporters exhibited a significant increase in their abundance throughout the course of osmotic stress. These antiporters are known to be implicated not only in osmotic adaptation, but also pH homeostasis [63].

Apart from the expression of genes and proteins for the uptake of several compatible solutes as already mentioned, exposure of *E. coli* to a sudden downshift in a_w_ also caused a significant alteration in the activity of other transport systems. It was observed that abundance of several transport proteins for phosphate and amino acid was induced. These included the proteins involved in the uptake of arginine (ArtQMP; at 310 min of the treatment) and methionine (MetQN; at all time points). For the group ‘peptide and nickel transporters’, production of SapCF, which are components of the SapABCDF uptake system for peptides, was induced at 30 and 310 min after the a_w_ downshift. Proteins involved in the transport of murein oligopeptides (OppBD) also exhibited a significant increase in expression at time 310 min.

The transcriptomic analysis revealed a significant down-regulation of several genes, encoding transporters for minerals, and inorganic and organic ions, such as sulfate/thiosulfate (*cysWP*; at 80 min after a_w_ downshift) and molybdate (*modABC*; at 310 min). Transcription of the *potAB* operon involved in the uptake of polyamines, putrescine and spermidine was also reduced at 80 and 310 min following the shift. This may indicate a decrease in the cellular level of polyamines. Lack of polyamines has previously been shown to be linked to abnormal growth and oxidative stress-induced damage [64]. Furthermore, genes encoding monosaccharide and oligosaccharide transporters were found to be up-regulated. These included those responsible for the uptake of xylose (*xylFHG*; at 80 min) and maltose (*malEGFK*; at 310 min). It has previously been reported that catabolism of maltose can contribute indirectly to enhance the levels of endogenously synthesized osmolytes such as glutamate and *N*-acetylglutaminylglutamine amide, facilitating growth at elevated osmolarities [65].

Increased production of several proteins associated with the uptake system for metallic cations, iron-siderophores and vitamin B12 was observed in response to osmotic upshift. TonB and ExbBD, which make up the TonB-ExbBD energy transducing system to provide the energy source required for the uptake of iron-siderophore complexes and vitamin B12 across the outer membrane, were significantly up-regulated throughout the treatment. This was accompanied by the observations that FhuCD were significantly up-regulated (4.42 log_2_-fold on average) at all time points, indicating increased activity of the TonB-dependent iron (III) hydroxamate transport system. Abundance of FepA, a subunit of the TonB-dependent uptake system for ferric enterobactin also appeared to be significantly induced (2.63 log_2_-fold on average) within the first 30 min of the shift. In keeping with these findings, the response of *E. coli* during steady-state growth at a similar stress condition (25°C a_w_ 0.967) revealed an increase in overall abundance of proteins involved in the transport of cations and iron compounds [9]. The enhancement of iron uptake observed here may be associated with an increase in levels of intracellular hydrogen peroxide (H_2_O_2_) and superoxide (O_2_^-^), which invoke the oxidative stress response [62]. This also corresponds well with the present observations in which a number of genes and proteins involved in defense mechanisms against oxidative stress were significantly up-regulated by osmotic stress (see below). In addition, BtuE a component of the TonB-dependent vitamin B12 transport system exhibited a significant increase in its abundance of up to 2.45 log_2_-fold within the first 80 min of the stress.

### Fatty acids and lipids metabolism

*E. coli* membranes typically comprise three major phospholipids, including phosphatidylethanolamine (PE), phosphatidylglycerol (PG) and cardiolipin (CL). Growth at low a_w_ typically leads to changes in membrane lipid composition by increasing the proportion of anionic (PG and CL) over zwitterionic (PE) phospholipids. Such changes are to prevent the membrane lipids from adopting a non-bilayer phase, which otherwise could disrupt membrane function [66]. However, neither transcriptomic nor proteomic data provided strong evidence supporting this adaptive strategy. A significant increase in overall abundance of enzymes in the biosynthetic pathway of phospholipids was observed within the first 30 min of imposition of the osmotic stress (Table S4 in File S1). Phosphatidylserine synthase (PssA) and cardiolipin synthase (Cls) were found to be significantly up-regulated (2.94 log_2_-fold on average). PssA and Cls proteins are responsible for the *de novo* synthesis of PE and CL, respectively, suggesting that increased production of these phospholipids occurs. Despite that the physiological role of PssA under osmotic stress is unclear, PE has previously been shown to be important for acid adaptation [67].

Hyperosmotic stress was found to have no significant effects on overall expression of genes and proteins involved in the major pathways for fatty acid biosynthesis (Table S4 in File S1). This was despite the observation that the abundance of FabZ was significantly induced by up to 4.03 log_2_-fold at all time points of the stress. The FabZ enzyme plays an important role in the biosynthetic pathways of both saturated and unsaturated fatty acids. In contrast, the transcriptomic analysis revealed a significant up-regulation of the *cfa* gene, which encodes cyclopropane fatty acyl phospholipid synthase at time 80 and 310 min. The increase in cyclopropane fatty acid content in the cell membrane has previously been demonstrated to assist cells to maintain intracellular pH homeostasis by reducing membrane permeability to protons. This lipid modification provides protection against acid stress and other stress conditions such as high salt concentration and ethanol [68]–[70]. Furthermore, our observations on fatty acid composition are consistent with the study of Guillot et al. [71] on the response of *Lactococcus lactis* to osmotic stress, in which that organism was reported to increase the level of cyclopropane fatty acids, whereas the unsaturated-to-saturated fatty acids ratio remains unchanged.

Several genes, encoding the key enzymes for lipopolysaccharide biosynthesis (i.e. *lpxH* and *waaA*) were down-regulated with a significant negative *T*-value at 80 and 310 min after hyperosmotic shift (Table S4 in File S1). The apparent reduction of lipopolysaccharide biosynthesis might indicate that outer membrane instability occurs during adaptation to hyperosmotic stress. Consistent with this, previous studies have reported that a defect in lipopolysaccharide biosynthesis leads to the lack of a continuous lipopolysaccharide layer in the outer membrane, causing increased susceptibility of bacterial cells to hydrophobic antibiotics [72], [73]. Furthermore, it has been demonstrated that the envelope stress caused by the defective biosynthesis of lipopolysaccharide increases the biosynthesis of the exopolysaccharide colanic acid [74], [75]. This, indeed, agrees well with earlier observations, indicating that the Rcs system-regulated colanic acid biosynthesis becomes activated (see below).

### Cell structure components

In response to hyperosmotic stress, *E. coli* increased expression of several genes and proteins involved in the Rcs phosphorelay system that regulates the biosynthesis of colanic acid [32] (Table S5 in File S1). The T-profiler results revealed that a significant increase in overall expression of genes known to be induced by Rcs regulon [32] occurred from 30 min of the osmotic treatment onward, whereas several Rcs-dependent proteins were significantly up-regulated only at the time point at which *E. coli* had resumed growth (i.e. at time 310 min). These genes and proteins were also found to be amongst the most highly up-regulated in the present study (Table S2 in File S1). Consistent with these findings, Kocharunchitt et al. [9] also demonstrated strong up-regulation of Rcs-dependent elements together with a high level of colanic acid production in *E. coli* cells during steady-state growth under a similar stress condition (25°C a_w_ 0.967). The importance of colanic acid has frequently been described as protecting cells against a variety of stresses, including osmotic stress [76]–[78], and has been shown to be involved in biofilm formation [79]. Although the physiological role of colanic acid is not well understood, it is thought that colanic acid expressed on cell surfaces simply provides a physical barrier to protect cells from hostile environments [78], [80]. Allen et al. [81] has reported that colanic acid confers a strong negative charge to the cell surface. This negatively charged cell surface has led to the suggestion that colanic acid may help *E. coli* to maintain hydration of the cell surface, and to preserve the membrane lipids in the proper bilayer phase, as part of the adaptive strategies in response to such stress [65], [66]. However, the findings of Kocharunchitt et al. [9] indicated that colanic acid is not required for growth and survival under osmotic stress. A comprehensive understanding on the function of colanic acid is, therefore, needed to understand the induced activity of the Rcs system-controlled colanic acid biosynthesis under osmotic stress.

### Virulence properties

Adaptation of *E. coli* to high osmolality appeared to be associated with increased virulence. It was evident in the transcriptomic analysis that several genes encoding virulence factors in the locus of enterocyte effacement (LEE) pathogenicity island (PAI) were significantly up-regulated. These included *ler* encoding a regulator required for activation of LEE (from 80 min of the stress onward), *eae* encoding the outer membrane adhesin intimin (only at time 80 min), *tir* encoding the translocated intimin receptor (from 30 min onward), and *esc* operon encoding major components of type III secretion system (only at time 310 min). In contrast, the proteomic data did not provide strong evidence of increased abundance of virulence proteins (Table S2 in File S1). The observed increase in transcript levels of virulence genes, however, supports the idea that bacteria typically enhance their virulence after exposure to environmental stresses [6].

### Bacterial chemotaxis and motility

An immediate increase in overall expression of genes and proteins involved in chemotaxis and motility was observed after osmotic upshift, as indicated by significant positive *T*-values (Table S3 in File S1). This was followed by a general decrease in their level over the course of osmotic treatment. Accordingly, our transcriptomic data revealed a significant down-regulation of *rpoF*, which encodes a sigma factor responsible for controlling the transcription of genes involved in chemotaxis and motility [6], at 80 and 310 min of the treatment. It was also found that chemotaxis and motility genes and proteins were amongst the most heavily down-regulated in osmotically-adapted cells (i.e. at time 310 min) (Table S2 in File S1). Kim et al. [82] has also reported a transient increase in expression of flagella biosynthetic elements in response to osmotic shock. This induced activity may be an initial response for cells to move away from osmotic stress [83]. Furthermore, the strong down-regulation of chemotaxis and motility systems observed here supports recent suggestions that these systems are the most dispensable functions during steady-state growth at high osmolality (25°C a_w_ 0.967) [9]. This adaptive response of *E. coli* is in keeping with the fact that expression of genes encoding flagella are negatively controlled by the master regulator RpoS [29], and the Rcs system-activated colanic acid biosynthesis [84].

### General stress response

Exposure of *E. coli* to a sudden downshift in a_w_ resulted in induced activity of the master stress regulator RpoS. This was evident in the transcriptomic data in which the mRNA level of *rpoS* significantly increased up to 3.40 log_2_-fold from 30 min of the shift onward, whereas the proteomic analysis revealed a significant up-regulation of RpoS only at time 80 and 310 min (4.66 log_2_-fold on average). Accordingly, the T-profiler analysis of both datasets showed a large increase in expression of several genes and proteins previously known to be induced by the RpoS regulon [29]–[31] over the course of treatment (Table S5 in File S1). Many of these have been described to be involved in osmoprotection [4], [51], including osmotically inducible proteins (*osm* operon; at 30, 80 and 310 min of the treatment); *dps*/Dps, which is responsible for the protection of DNA (at 80 and 310 min); *otsAB*/OtsAB, which are involved in the *de novo* synthesis of trehalose (at 80 and 310 min); and a group of elements with undefined functions (*elaB*/ElaB, *yciEF*/YciEF, *ygaM*/YgaM and *yjbJ*/YjbJ; at 80 and 310 min). The transcriptomic study also found that a number of RpoS-dependent genes, which are known to be associated with defense mechanisms against other stresses [31], [85], increased expression at 80 and 310 min of the treatment. These included genes within the acid resistance fitness island (*gadABWX* and *hdeBD*), and those involved in response to oxidative damage (*katE* and *sufABD*). The proteomic analysis, however, revealed up-regulation of proteins involved in acid (GadAB; 2.47 log_2_-fold on average) and oxidative stress responses (KatE and SufAD; 5.35 log_2_-fold on average) only at time 310 min. Taken together, these observations support previous reports, demonstrating that the general response network established by the RpoS regulon typically provides cross-protection against diverse stress conditions [9], [29]–[31].

Apart from activation of the RpoS regulon as described above, *E. coli* cells transiently induced additional stress responses or those elements controlled by other regulons (i.e. the RpoE and RpoH regulatory systems) during adaptation to hyperosmotic stress (Table S2 in File S1). A significant up-regulation of *rpoE*/RpoE was observed at 30 min (3.19 log_2_-fold; in the proteomic analysis) and 80 min (2.45 log_2_-fold; in the transcriptomic analysis) of the osmotic stress, while *rpoH* expression significantly increased (1.81 log_2_-fold on average) at time 30 and 80 min. The RpoE and RpoH regulons are known to be responsible for the control of protein misfolding in the cell envelope and cytoplasm, respectively, and it has been reported that the transcription of *rpoH* is RpoE-dependent [28], [86]. Furthermore, the majority of genes whose expression is under the control of RpoH were found to be significantly up-regulated during the recovery period after hyperosmotic shock (i.e. at time 80 min). These included those encoding molecular chaperones (*dnaK, htpG* and *ibpAB*; 2.51 log_2_-fold on average), which function in facilitating protein re-folding and stabilization, and preventing aggregation of misfolded proteins. DnaK is a member of the major DnaK/DnaJ/GrpE chaperone system, and has previously been reported to be involved in adaptation to osmotic and oxidative stresses [52], [87]. Although other chaperone proteins (i.e. HtpG and IbpAB) have only minor activity in *E. coli* [88], previous studies have suggested that they play a significant role in *de novo* protein folding under stressful conditions, presumably by expanding the activity of the major system to interact with newly synthesized polypeptides [88], [89].

The apparent response of *E. coli* cells to osmotic upshift through a transient induction of the RpoE and RpoH systems, and activation of the RpoS regulon reflects their robust ability to activate multiple defense mechanisms, and may also underlie the observation of a complex pattern of microbial growth behavior in response to such stress. In support of this, it has previously been suggested that increased activity of the RpoE and RpoH regulons may serve as a multipurpose emergency response to repair protein misfolding and facilitate the proper folding of newly synthesized proteins after loss of turgor (as would be expected during the injury and recovery period), while simultaneously activating the master stress regulator RpoS to mediate long-term adaptation at high osmolality [9], [89].

The present study provides evidence suggesting that *E. coli* might form filaments during adaptation to an abrupt downshift in a_w_. It was observed in the transcriptomic data that expression of *sulA* encoding SOS cell division inhibitor was induced by up to 3.65 log_2_-fold from 30 min of the shift onward. This was accompanied by up-regulation of gene encoding the cell filamentation protein (*fic*) from 80 min onward (1.74 log_2_-fold on average). The products of both genes have previously been demonstrated to play an important role in cell filamentation [90], [91]. Additionally, the apparent increase in *sulA* expression suggests that DNA damage might occur as a result of osmotic stress. This is in line with the previous findings of Fitt et al. [92] and Ribeiro et al. [93].

It is noteworthy that *E. coli* exposed to an hyperosmotic shift did not show a strong alteration in expression of genes and proteins involved in the biosynthesis of alarmone (p)ppGpp that instigates the stringent response [94] (Table S2 in File S1). This was despite that the proteomic data revealing a significant increase in abundance of SpoT (2.08 log_2_-fold) only at 310 min of the shift. However, previous studies have reported that the mechanism of (p)ppGpp action is typically characterized by a major change in global expression with the negatively controlled elements involved in cell growth (i.e. translation processes) and the positively controlled elements involved in amino acid biosynthesis, transcriptional factors, or alternative sigma factors (i.e. RpoE, RpoH and RpoS) [94], [95]. This indeed corresponds well with the present observation on the response of *E. coli* to hyperosmotic stress as described above, suggesting that the stringent response might be induced under such stress.

## Conclusion

The present study provides a deeper insight into the growth kinetics and potential mechanisms enabling survival and growth of *E. coli* O157∶H7 Sakai when subjected to a sudden downshift in water activity. Such knowledge will be used to develop more targeted approaches for the food industry to eliminate or control this pathogen.

## Supporting Information

File S1 Table S1 Summary on % false-positive discovery rate calculated for each MudPIT run. Table S2 Comparison of differentially expressed genes and proteins in *Escherichia coli* O157∶H7 Sakai during exposure to hyperosmotic shift. Table S3 T-profiler analysis of transcriptomic and proteomic data based on JCVI CMR functional categories. Table S4 T-profiler analysis of transcriptomic and proteomic data based on Ecocyc and KEGG functional categories. Table S5 T-profiler analysis of transcriptomic and proteomic data based on major regulons.
(XLSX)
